# Book Reviews

**Published:** 1819-10

**Authors:** 


### CRITICAL ANALYSIS

OF

### RECENT PUBLICATIONS, IN THE DIFFERENT BRANCHES OF MEDICINE AND SURGERY;

### SELECT MEMOIRS, AND HISTORIES OF CASES;

### In the Literature of Foreign Nations.

Πατρίδας ἄρα
Ανδράσιν, ἃ πατρὰς ἄνδρες, ἀγαλλόμεθα.

***Discourses on the Elements of Therapeutics and Materia Medica.*** By NICHOLAS CHAPMAN, M.D. Professor of the Institutes and Practice of Physic and Clinical Practice in the University of Pennsylvania; President of the Philadelphia Medical Society, &c. &c. 2 vols. 8vo. pp. 377 and 494. James Webster, Philadelphia; and Souter, London. Vol. I. 1817; Vol. II. 1819.

"To communicate what I have tried, and leave the rest to others for farther enquiry, is all my design in publishing these papers."—NEWTON.

THERAPEUTICS, although the branch of medical knowledge the earliest cultivated, and the true object and end of all our researches, is, as a science, the most imperfect and most enveloped with the illusions of error. Methodists, dogmatists, and eclectics, in all ages, however ardently devoted to their respective theories and haughty and authoritative in their schools, have, on coming to the bedside of the patient, bowed their heads to the empirics, and been content to use their remedies under the guidance of their instructions.

"ANDROMACHUS (says BORDEU) effected a *chef-d'œuvre* necessary to the human species, when he imagined, or when he collected, the materials of the *Theriaca.* He would be overwhelmed with ridicule, were he to come amongst us, and attempt to reply to the theoretical objections that might be made to his composition: he would not be received a bachelor in our schools: but his remedy is every-where resorted to with eagerness. I have for many years seen a bolus of theriaca given every night to all the patients in the hospital at Montpellier, whilst the schools in that metropolis of medicine resounded with invectives against the composition."

These remarks are, to a certain extent, applicable to the whole of the materia medica.

In order fully to expose the cause of this vagrancy of human conduct, it would be necessary to determine why the most fervid speculators in morals as well as in physics, in the midst of the imagined glory about to surround them from the application of their Utopian schemes, are content, when they come amongst us, to conform to social order and to walk in the common train; and why Pyrrho, who doubted of his own existence, should quarrel with his sister about some trivial domestic affairs.

This is one of the chief causes of the little progress of true knowledge in therapeutics, and of the facility with which it has been permitted to be encumbered with errors. Another source has existed, even in the conduct of those men of science who have laboured for its improvement and the extension of its bounds. This has been evinced in a want of accuracy in determining the precise relations between causes and effects; and from *post hoc ergo propter hoc,* having been a maxim commonly applied. Even Robert Boyle believed that *amulets* might cure diseases by their physical agency, and reasoned on the manner in which it might be effected.

Another cause why rational knowledge of this subject is so confined, has been the separating the study of the properties and qualities of medicinal substances from that of their effects under the different forms of disease; and especially in neglecting to combine it with pathology.

Therapeutics, and the knowledge of the qualities of the materia medica, are so inseparably connected, that it is impossible to reason on either of them in a distinct point of view; and, without pathology, reasoning on them must be devoid of results worthy of an enlightened age.

After M. Alibert, who has so well developed the sketch drawn by Bichat, Dr. Chapman is the first who has comprehensively treated the subject in the methodic manner to which we have alluded. There wanted but originality in the plan of his work, to give the author a claim to the introduction of a system calculated in an eminent degree to favour the progress of medicine; yet it seems apparent that Dr. Chapman is not, in this respect, an imitator: he has not noticed the labours of those who have preceded him in the same course; and the statement made in the preface to this work, would render it probable that he was not acquainted with them. He was called, unexpectedly, in the year 1813, to teach the materia medica in the university of Pennsylvania, without having studied the subject with that precise and definite view. After having given three courses of lectures, he was transferred to the chair of the institutes and practice of medicine in the same school; and he was then so urgently solicited to publish his Lectures by the students, as to be induced to offer them to the public, and without having had leisure to make any other alteration in them than the revision of a few passages.

The author commences with a brief sketch of the history of the materia medica, which should have been either more perfect or altogether omitted; since he has not noticed, amongst others of less importance, the labours of Geoffroy, Vogel, Fourcroy, Arnemann, and Darwin; nor even those of later authors, whose doctrines are in some respects similar to his own,—as Alibert, Kretschmar, and his compatriot Dr. Bibb; and he has referred to those of several physicians not immediately connected with it. Proëmia of this kind may do for the school, but beyond that they should not extend.

Dr. Chapman then adduces some reflections on "the improvement of the materia medica." These are judicious, but not sufficiently novel to detain us in our progress to the more interesting and valuable matter contained in the body of the work; and, before we can arrive at that, we have a long and arduous task to perform, in following the author in his course to the promised Latium.

On a subject hitherto so imperfectly explored, at least in a scientific manner, and so deficient in accurate observation as "*the modus operandi of medicines,*" speculations extending beyond the boundaries of precise induction, could be with difficulty avoided in an attempt to form a general theory; especially when guided by an enthusiastic spirit, impatient of intervening obstacles, and determined to avert the perplexities of doubt. Thus, we find in this part of the work of Dr. Chapman, an inclination to hypothesis rather than to theoretical reasoning; and a disposition to consider many points as finally determined, which yet remain to be decided by the results of further researches.

These, however, are errors which almost necessarily spring from the circumstances under which the work was formed. An ardent mind possessing a multitude of original conceptions, adapted, if true, to effect a revolution in the doctrine of the subject to which they relate, does not willingly abandon them; especially when they glow with the halo with which genius will envelop even the offspring of error. Much time and reflection are necessary to lead to the resignation of some, and to doubts respecting the validity of others; and this time and opportunity for reflection were not possessed by Dr. Chapman. But he will not, we trust, finally resign his doctrine without further consideration; for, if erroneous, it must, in its application to the art of medicine, prove injurious; and his talents, and his dignity as a professor, may give to it an unlimited authority over the minds of many of the pupils of his school.

"My theory of the operation of medicines," says Dr. Chapman, "is of modern date, and alleges, that they all act by exciting a local impression, which is extended through the medium of sympathy. By many, however, it is still believed that certain articles, at least, enter the circulation, and produce their effects in this way."

This is one of the points on which, it appears to us, the author has made an erroneous, or at least premature, decision: for, that certain substances will, when admitted into the circulation, produce their proper effects, is proved by experiment. The admission of extraneous matters into the circulation, will be hereafter considered. The circumstance we have above stated, is especially shown by the experiments of Mr. Brodie and M. Magendie with some of the vegetable poisons. M. Magendie separated the limb of a dog from its attachment to the rest of the body, except by that of the artery and vein: these vessels were then divided, and afterwards joined by the medium of pieces of quill. Some of the upas antiar poison was then inserted into the paw of this limb; which was followed by the death of the animal, as speedily as if it had been unmutilated,—that is, in about four minutes. This shows that the poison may exert its agency through the medium of the blood. Viewing the subject in another aspect, it appears from the experiments of Mr. Brodie, that, by placing a ligature on the vessels, leaving the nerves at liberty, no apparent influence on the system took place from the introduction of the same poison into the leg whilst the obstruction to the circulation remained; though, on removing this after an hour, the animal soon began to experience its effects, and died in about twenty minutes. It may here be said, that stopping the circulation in the part destroyed its vitality; and this objection is sufficiently well-founded to prevent any conclusions to be formed from this experiment in opposition to the doctrine of sympathy, when considered abstractedly; but it may be worthy of some consideration, in conjunction with the experiments of M. Magendie.*

But, neither these, nor any other observations or experiments with which we are acquainted, absolutely refute the opinion of Dr. Chapman, that medicines, as ordinarily applied to the intestines and skin, produce the effects we witness, and attribute to them, only by sympathy. Let us, then, examine his arguments in favour of his doctrine.

"To reach the circulation," says Dr. Chapman, "medicines must pass either by the lacteals or lymphatics. Now, it seems more than probable, in either case, their powers would be so neutralized by the preparatory processes of animalization, as to be deprived of all activity."

The latter notion is refuted by the observations made by Sir Everard Home, in his experiments to determine the functions of the spleen. Rhubarb taken into the stomach, imparted to the blood and the urine its peculiar odour and colour; turpentine also, taken internally, gives to the urine its proper odour. We shall hereafter have occasion to recur to this subject.

Dr. Chapman argues in favour of his opinion from the apparent identity of the nature of the chyle, whether obtained from vegetable or animal substances. This is not determined with sufficient precision to admit of his inferences. That the absorbents of the intestinal canal will transmit fluids possessing properties different from those of the chyle, is proved by water being taken up from it; and we have no facts to show that fluids of more stimulating, as well as those of less stimulating, qualities, may not be admitted by those vessels. May not, too, different sets of those vessels,—that is, both lacteals and lymphatics,—spring from the cavity of the intestines?

But Dr. Chapman attributes a *digestive* power to the whole lymphatic system. He says,

"No one, who has carefully attended to the phenomena of the absorbent system, can help admitting, that every section of it is endowed with the power of *digestion* and *assimilation*; and the lymphatics quite as conspicuously as the lacteals. This capacity is given, as a provision of nature, to exclude noxious matters from the circulation.

"The absorbents, in most instances, are fully adequate to this end; and when they are not, the substance penetrates to the first conglobate gland, which takes on inflammation, and arrests its further progress: these organs acting here as centinels, guarding the exterior approaches of the body. That some of the properties of certain articles, as the

* These experiments were related to the *Institute* of France, and the *Royal Society* of London; and are published, respectively, in their *Reports* and *Transactions*.

odour of garlic, and the colouring matter of madder, are displayed in the secretions and excretions, I am not disposed to deny. But it does not hence follow, that these substances entered the circulation in their primitive state. Directly the reverse, indeed, seems to be proven; as neither the one nor the other can be detected in the serum of the blood."

All this is refuted by the observations of Sir Everard Home, on the occasion to which we have already alluded.* To account for the substances above designated, in the *urine,* Dr. Chapman says, when they are "thrown into the secretions or excretions, being removed beyond the sphere of the vital energies, the chemical affinities are sometimes again brought into play, by which these substances are in part, or wholly regenerated." This, in itself, is a strange effort of the imagination to support an hypothesis; but it will not account for the colour and odour of rhubarb in the blood.

"Whether this explanation be received or not," continues the author, "it must at least be acknowledged, that no substance, in its active state, does reach the circulation; since experiments have shown, that a few drops even of the mildest fluid, as milk or mucilage, oil or pus, cannot be injected into the blood-vessels, without occasioning the most fatal consequences."

This statement is contrary to that of some of the most eminent physiologists on this side the Atlantic; and, unless we had a particular account of those experiments to judge from, we know not what importance to attach to them; especially when it was ascertained by Bichât, and has been also proved by other physiologists, that a small quantity of air thrown into the blood-vessels will cause death, apparently from its effects in the brain; and, if much care be not taken in using the common syringe in performing those experiments, an introduction of air into the vessels must take place. In some recent experiments by Dr. Blundell, air, to the quantity of five drachms by measure, was thrown into the femoral vein of a dog, at short intervals, without producing death; but it caused very severe distress, palpitation of the heart, tremors, difficulty of breathing, &c.: the fact that death will commonly ensue from the introduction of a small quantity of air into the circulation, is not invalidated by them.†

Let us proceed in the consideration of the author's arguments. He says,

"Conceding, however, to the humoral pathologists all which their doctrine demands, and still innumerable difficulties remain in the way of its adoption, to account for the operation of medicines. Not to

---

* See *Philosophical Transactions*, an. 1807.

† On this occasion we cannot refrain from the indulgence of citing one of the most useful reflections of our literary Aristarchus:

"Even a man whose genius qualifies him for great undertakings, must be content to learn at least from books the present state of human knowledge, that he may not ascribe to himself the invention of arts generally known; weary his attention with experiments, of which the event has been long registered; and waste, on attempts which have already succeeded or miscarried, that time which might have been spent with usefulness and honour upon new undertakings."

dwell tediously on this subject, I shall content myself at present with merely mentioning that we are not at all informed by it, why our remedies, after mixing with the blood, should be directed to one organ in preference to another, as mercury to the salivary glands; or how, indeed, they operate at all. Nothing, surely, is less probable than that dead matter, by mere mixture with an insensible fluid like the blood, should produce any effect on a living system. If it be alleged, as it has sometimes been, that the action of medicines, under such circumstances, is on the surface of the blood-vessels, then the doctrine becomes utterly deserted, and we are forced to recur to sympathy, as affording the only explanation."

Some ingenuity is here exerted to render obscure what is really clear and intelligible with the utmost facility. We shall first refer to a fact, which is better than any logical arguments, to show that dead matter mixed with the blood will act on the living system: the experiment of M. Magendie with the upas poison already related proves that. In order to understand the operation of certain medicines on certain organs especially, it is not necessary to suppose that they should be directed to one organ in preference to another. Each medicinal substance has its peculiar quality, which is irritant with respect to the animal economy; and each organ has its peculiar sensibility. These are propositions which, we believe, will be universally admitted. It is, then, a rational induction to suppose that those substances, the properties of which bear a certain relation to certain organs, will affect them; whilst those whose irritating quality is below such a degree of relation, will have no sensible influence on them. Thus, there are many substances which produce sensible effects on every part of the system; and these are generally called *acrid* to the *taste:* their irritating qualities are so great, as to bear a high degree of relation to the sensibility of every organ. There are others which affect only particular parts, and these are generally more or less *insipid:* their irritating qualities are so low, as to bear the relation necessary to produce sensible effects only to certain organs, and, consequently, the parts sympathising with them. This is only a general view of the subject: the particular laws are probably to be sought for in those which cause the parotids to secrete the saliva,—the liver bile, &c.* In the present state of our knowledge, perhaps nothing more than an hypothesis can be formed; but that which is more nearly allied to theory, and is best supported by analogy, should be considered as the most valid. We are however not obliged, by the laws of argument, to prove the converse to the propositions of an adversary, when they are in opposition to the doctrines generally received; though such a demand is at last made by Dr. Chapman. For, after having cited from Mr. Murray the observation, that the effects of many substances are produced in a shorter time after they have been received into the stomach, than they would be were they to act by being absorbed with the chyle into the circulating mass, and therefore act by the "medium

* See the extract from the works of Bordeu in the *Collectanea* of the present Number: it is from that we have deduced the above notions.

of nervous communication," he says, "The principle being thus clearly established in so large a number of instances, which, if necessary, might be still further increased, it appears to me, that it should be admitted as an universal law, unless exceptions to it are very clearly made out and demonstrated." This, in scholastic disputations, would be considered as the signal of a defeat; and Dr. Chapman here begs the question, without having produced any valid arguments to show his claims to it. Because some remote effects of several medicinal substances depend on sympathy, we cannot thence rationally consider that as the absolute law of their agency.

"To multiply causes superfluously," Dr. Chapman says, "is against one of the fundamental rules of philosophizing, and is not less repugnant to the general course of nature, whose means are proverbially distinguished by great simplicity and uniformity." It is this disposition to generalization that has been the origin of the greatest and most numerous errors with which the history of the opinions of men abounds. On the other hand, to contemplate every phenomenon individually, though it may not be attended with a rapid progress in knowledge, will certainly not lead to error. We know nothing of the course of nature but what we witness in its phenomena; and this simplicity and uniformity of them has not been established by philosophers. We must not deviate from the course of induction; if we do, we are more likely to enter one of the numerous paths of error, than the single one of truth.

Let us proceed to the remainder of the author's arguments.

Dr. Chapman says, he can adduce the most decisive evidence, from experiments conducted by himself and Doctors Physick and Seybert, that none of the preparations of mercury, iron, copper, lead; nor the colouring matter of indigo, of madder, or of rhubarb; can be traced even so far as the chyle. This statement is in opposition to the results of accurate observations and well-conducted experiments by several of our best physiologists; and, without a detail of those to which Dr. Chapman alludes, we certainly cannot consider them as controverted. The circumstance of persons taking mercury in considerable quantities having coins, &c. worn about them coated with that metal, is familiar to every medical practitioner, and is a forcible argument against the assertion of Dr. Chapman. We have already referred to evidence of the existence of rhubarb and madder in the serum of the blood.

Dr. Chapman notices the operation of poisons on animals from which the heart has been removed, in support of his doctrine; and we readily admit that it is valid to a certain extent: but this is not a sufficient basis for the foundation of an opinion, that such is the absolute law of the operation of medicines. We cannot, except by sympathy, understand how the application of a drop of prussic acid, or of essential oil of bitter almonds, produces instant death on being applied to the tongue, &c. of many animals. And it seems also necessary to refer to this, to explain the occurrence of salivation from a single application of a dressing of mercurial ointment to a small wound, or from a grain of calomel. These, and many other observations familiar to medical practitioners, must probably be explained by the agency of that law.

The alimentary canal, the surface of the body, and the organ of smell, are designated as the parts " on which remedies, and perhaps the causes of disease, more especially operate ;" and the two former are considered to sympathize with the whole of the rest of the economy.

It was long since ascertained, that a great proportion of the materia medica will produce the same effects on the system when applied to the skin, as when taken into the stomach ; but it has been supposed that they were carried into the circulation by cutaneous absorption. Dr. Chapman considers that this is rendered doubtful, by some well-conducted experiments ; especially those made by M. SEGUIN and Dr. ROUSSEAU. By cutting off all communication with the lungs, the latter physician found, that, though the surface of the body were bathed with the juice of garlic or the spirit of turpentine, none of the qualities of these fluids could be detected, either in the urine or the serum of the blood. The substances here mentioned are evidently objectionable ones for the decision of this question; and that some vegetable substances may be absorbed from the surface of the body, has been demonstrated.

" Determined, if possible, to put this agitated question to rest, Dr. Rousseau, assisted by his friend Dr. Samuel B. Smith, has subsequently performed a series of experiments; many of which I witnessed," says Dr. Chapman, " and can therefore bear testimony to their accuracy, with every variety of substance, mild and acrid, volatile and fixed, nutritive, medicinal, and poisonous. The result of these extensive researches, is,

" 1. That, of all the substances employed, madder and rhubarb are those only which affect the urine; the latter of the two the more readily enters the system. Neither of these articles can be traced in any other of the secretions or excretions, or in the serum of the blood.

" 2. That the power of absorption is limited to a very small portion of the surface of the body. The only parts, indeed, which seem to possess it, are the spaces between the middle of the arm and shoulder."

Having shown the incorrectness of some of the other statements of this kind, we may be readily allowed to doubt of the accuracy of those just adduced. The observations in the second paragraph, too, are opposed by the results of analogous experiments made by Dr. SEWELL, who found that madder was detected in the urine, after having placed his feet only in a decoction of that substance.

Until, then, the old doctrine, that some medicinal substances act on the system through the medium of the fluids, is refuted, or a more satisfactory one given in its place, we must continue to profess it. Neither of these, we think, has been effected by Dr. Chapman ; though we believe the following paragraph describes some important truths, which merit the assiduous attention of the medical practitioner. The doctrine naturally flows from the physiological principles of BICHAT.

" Conformably to the theory which I have proposed, whenever a medicinal substance is applied to a susceptible portion of the body, externally or internally, an action is excited, which is extended more or less, according to the diffusibility of the properties of the substance, or degree of sympathetic connexion which the part may maintain with the body generally. Thus a set of actions is raised, every one of which is precisely similar, provided they are confined to the same system; by which is to be understood, parts of an identity of structure. If, however, the chain runs into other systems, it loses its homogeneous character; the actions being modified by the peculiar organization of the parts in which they may take place. These are principles of universal application. In every case, whether it respects the operation of remedies or the production of disease, the spot primarily acted upon is a point, from which is diffused the radiated impressions."

[*To be continued.*]

---

### *An Account of the Structure of the Brain and its Appendages.*

By Professor Lauth.

[*Continued from page* 258.]

§ I. The cerebrum. 1°. *The peripherical arrangement.*—This arrangement comprises the hemispheres, the convolutions, and the cavities of the cerebrum, as well as the choroid plexuses.

A. *The hemispheres.*—The cerebrum bears a resemblance, in its external form, to the cavity in which it is contained. Above, it is, like the cap of the cranium, rather uniformly convex; but beneath, or at its base, it presents an unequal surface, modelled on those of the foss which receive it. Its anterior extremity, although not very acute, is yet less obtuse than its posterior. It is divided by the falx of the dura mater in two lateral portions or *hemispheres*, themselves subdivided into three *lobes*, anterior, middle, and posterior. The anterior and posterior lobes are completely separate from above downwards; but the middle, after a deep division, are united in the middle by the corpus callosum. The anterior and middle lobes are, besides this, distinguished one from the other by a deep groove, termed the *fissure of Sylvius*. The middle and posterior lobes are not so exactly separated; for the furrow that Malacarne* and Vicq-d'Azyr† observed, is not constant. It is for this reason that Haller‡ does not admit in each hemisphere more than two lobes, the one anterior, the other posterior, separated by the fissure of Sylvius. However, as the middle lobe is convex at its base and lodged in a deep fossa, and the posterior being flattened and placed on the tentorium of the cerebellum; and as, besides, the middle lobes are united at a certain depth, whilst the posterior are separate throughout their whole thickness; the division of each hemisphere into three lobes seems to be sufficiently proper. With regard to the term *hemisphere*, it is true, the form of the cerebrum is ovoid, and not spherical; and it is also true, that the base of this organ is not so rounded as its superior surface; yet the name of quadovoid, which might be substituted, not being very exact, it is better to preserve the denomination already received.

---

* *Encefalotomia*, P. ii. No. 9.

† *Trait. d'Anat. et de Physiol.* pl. xxv. f. 1, Nos. 4, 5, 6.

‡ *Elementa Physiol.* iv. 15.

B. *The fissures.*—The surface of the cerebrum has on its surface numerous tortuous grooves, which give to it some resemblance to the intestinal convolutions, in the direction of which no regularity has hitherto been discerned.* The pia mater gives out prolongations which enter these grooves, and, tracing their convolutions, is itself continued over both their surfaces. The most considerable amongst these grooves, is the fissure of Sylvius, which separates the anterior lobe, supported on the orbitary vaults, from the middle, lodged in the medial fossa, and of which Reil† has given a description under the name of the *valley*. The superior paries, which this author terms the roof of the valley, appertains to the anterior lobe; and the inferior is derived from the middle lobe. The form of the valley resembles an unequal tunnel in its cavity, surrounded by irregular convolutions.

C. *The lateral ventricles.*—After the external form of the cerebrum, we should examine its internal structure, to which the three openings mentioned in the preceding article will conduct us. But as, to discover it in this way, some preparation, not unattended with difficulty, is necessary, we shall rather arrive at it in the usual manner.

The hemispheres are removed by horizontal slices to the point of union of the middle lobes. To this effect, we part them before they are divided, that this union may be perceived, which is constituted by the *corpus callosum*, of which we shall hereafter speak. We then see that their internal and inferior edges lie at liberty on the corpus callosum, which is consequently larger than the interval of their internal surfaces. In proportion as we proceed in cutting from their surface, the white substance increases in extent; and, when they are removed to a level with the corpus callosum, the base, on which they were placed, represents a white floor, called the *centrum ovale* of Vieussens, divided into two lateral portions by the corpus callosum. If we now make an incision on each side at a little distance from the corpus callosum, we fall on a cavity, which is wholly discovered on breaking down the white substance with the handle of the scalpel, following the direction of that cavity.

The two cavities opened by this preparation, are the *lateral, tricornual*, or *anterior*, *ventricles*, to which the centrum ovale served as a roof, and which are separated by a septum pierced with a hole, by means of which they communicate. We regard in the ventricles, their flooring, their cavity, and their figure. The flooring is unequal, because of the tubercles that project in it, and which appertain to an arrangement of which we shall hereafter treat. Hardly any cavity exists in the state of health, because the roof is for the most part in contact with the floor, as is the case with the cavities of the mouth, the thorax, the abdomen, &c.; and during life the surfaces are preserved from the effects of friction by a vapour, which after death condenses into drops. Disease sometimes produces an effusion of serum, which renders the cavity more vast in proportion to the greater abundance of the fluid.

* Is it really true that the cerebral surface of some celebrated men has shown a regularity in the convolutions?

† *Archiv. für die Physiologie*, ix. 195.

Each ventricle has an irregular form, and is divided into three branches or *horns ;* one *anterior,* one *posterior,* and one *inferior.*

The anterior horn advances into the anterior lobe, very near to its frontal extremity, curving a little outwardly.

The posterior horn traverses the posterior lobe, sometimes to its extremity. It is at first turned convexly outwards, and ends by curving itself inwardly. We here perceive several grooves, and an eminence* between them, the whole of which has been compared to the paw of a cloven-footed animal, and which has been termed the *spur,*† *claw,* and *hippocampus minor.*‡

The inferior horn, particularly described by Tarin,§ Malacarne,‖ Vicq-d'Azyr,¶ and Wenzel,** is a curved canal, which descends a little backwards, then outwardly, towards the base of the cranium, where it turns immediately inwards, and terminates near the pons Varolii, between the crura cerebri and cerebelli, by an opening, by means of which, as we have already said, the internal surface of the brain is continuous with the external. There runs along the inferior paries of this horn a prominence called the *cornu ammonis,* which appertains to the arrangement of the corpus callosum, and which we shall hereafter describe. The cavity enlarges as it approaches the opening, and forms a sort of enclosure at the inferior portion of the hippocampus. According to Wenzel, this hippocampus communicates in the following manner with the external surface of the brain : The cerebral convolution situate at the middle of the internal edge of the base, takes a direction backwards toward the union of the crura cerebri and cerebelli ; a portion of this convolution forms the lobule termed *crotchet* by Vicq-d'Azyr.†† This crotchet unites with another portion of convolution, turns inwards, and communicates with the hippocampus.

D. *The plexus choroides.*—The *choroid plexuses* or *tela choroidinea,* are the vascular network extended on the pia mater, which glides from the surface of the cerebrum into its cavity. Of these, three are enumerated :

The *first* and the *second* penetrate, at the base of the cerebrum, by the opening there remarked, into the inferior horn of the lateral ventricle, the direction of which they follow. When they have arrived at the floor of the ventricle, each of them advances on the optic layer, and is directed towards the opening in the *septum lucidum,* where they meet and unite. Throughout their whole extent they furnish vascular filaments, destined as well for the department of the corpus callosum as for that of the crura cerebri, but of which the greater proportion appertain to the department of the corpus callosum ; so that the

---

* According to Wenzel, (*de Penit. Struct. Cereb.* 145,) this eminence bears a relation to the convolutions of the cerebrum.

† Malacarne, (*Encefalot.* ii. 75.)

‡ Vicq-d'Azyr, pl. vi. xv. No. 42.

§ *Adversaria Anat.* tab. iii.

‖ *Encefalot.* P. ii. No. 82.

¶ *Traite d'Anat. et de Physiol.* pl. xv. xvii. xxii. xxv.

** *De Penit. Struct. Cereb.* p. 136, 141.

†† *Traite d'Anat. et de Physiol.* pl. xvi. 14, 15.

choroid plexuses appear to belong principally to the latter department. These net-works often contain vesicles or hydatids. Wenzel* has described in them a bundle of vessels, containing graniform corpuscles, which he attributes to effused lymph.

The *third* choroid plexus insinuates itself below the posterior extremity of the corpus callosum, and advances along the vault as far as the opening in the septum lucidum, where it unites with the two former. It furnishes numerous branches to the *tubercula quadrigemina* and to the *pineal gland;* but the greater portion go to the corpus callosum.

2º. *Department of the corpus callosum.*—This central department, composed of the corpus callosum itself, of the septum lucidum, of the vault, of its pillars, of the mamillary eminences, of the cornu ammonis, of the posterior and inferior part of the middle lobe, and of the posterior lobe, is perceived when the hemispheres have been removed, and the lateral ventricles discovered.

A. *The corpus callosum.*—This name is given to the medullary band, situate from front to back in the middle of the centrum ovale. The interior edges of the hemispheres of the cerebrum cover its sides, and the falx is suspended on its middle part. The corpus callosum commences anteriorly at the distance of an inch from the frontal bone; and its posterior extremity, larger than the anterior, is about two inches from the occipital. It is about an inch in breadth, and situate lower than the centrum ovale; it is overrun, throughout the whole length of its superior surface, by two medullary cords, called the *longitudinal nerves of Lancisi*, separated by a groove, or *raphé:* this superior surface is also marked by transverse and superficial furrows.

The two extremities of the corpus callosum are differently formed. The anterior, very narrow, and placed between the inferior portions of the anterior lobes of the cerebrum, is turned from above downwards; whence an angle or fold results, which receives the anterior part of the septum lucidum, and terminates in a *beak.* The posterior, larger, forms a transverse *pad*, the inferior surface of which is continuous with that of the vault, and its angles are confounded with the cornua ammonis.

The corpus callosum, entirely composed of white substance, may be divided into two lateral portions. Wenzel asserts that this division may be observed in the fœtus until the age of seven months. It may be discerned also in the adult; for, on careful examination, we perceive the anterior extremity terminate by two beaks; and, on proceeding from this place, we may commence the division of the corpus callosum. Thus, the cerebrum is parted into two hemispheres, and these are united at their bases by means of the corpus callosum ; but this union is made by contact, and not by an intertexture of substance.

The incision made to penetrate into the lateral ventricles, serves also to expose the other portion of the department of the corpus callosum. On turning aside the divided edge of the centrum ovale, we

---

* *De Penit. Struct. Cereb.* p. 91.

discover, first, the inferior surface of this centre, and of the corpus callosum; then the profile of the septum lucidum, of the vault and its pillars, as it is represented by SÖEMMERING,* MEYER,† VICQ-D'AZYR,‡ and GALL.§ When we follow the method of Vicq-d'Azyr,‖ and cut the brain by horizontal slices from below upwards, we perceive the same parts in a front view.

B. *The septum lucidum.*—From the middle of the inferior surface of the corpus callosum, and from each of its sides, there descends, in a perpendicular direction, and anteriorly and posteriorly, a thin medullary lamina, which forms a transparent division between the two lateral ventricles. On a side view, this septum resembles a spheric triangle, the anterior side of which answers to the angle or fold of the corpus callosum, the superior to the inferior surface of that body, and the inferior to the floor of the ventricles. This figure depends on the corpus callosum not taking a horizontal direction, being depressed posteriorly, and the floor of the ventricles being elevated anteriorly, where it touches the corpus callosum. We may perceive the transparency of the septum, on carefully separating this small substance.

Between the two laminæ of the septum there is a little space called the *ventricle of the septum*, or the *fifth ventricle of the cerebrum*, which contains a vapour, and often some drops of liquid, which I have seen converted into an icicle. This ventricle varies in respect to size: I have seen it extend all along the superior edge of the septum, from the beak of the corpus callosum to its posterior extremity; and in other cases the laminæ of the septum have been attached so closely, that there existed hardly any vestige of a ventricle. MALACARNE¶ and WENZEL** have observed in its interior a little fossa at each extremity of the septum, and a canal directed from the anterior of those fossæ towards the third ventricle.

The septum lucidum is pierced with a hole, by means of which the three cerebral ventricles communicate. This hole was very early known, since MARCHETTI†† describes it, and it is represented by MONRO.‡‡ It is situate at the inferior edge, and towards the anterior extremity, of the septum; it is nearly two lines in diameter; and it permits the plexus choroides to pass from one ventricle to the other.§§

C. *The vault.*—The vault is formed by two small medullary bands, each of which is attached to the inferior edge of one of the laminæ of the septum lucidum which rest on the convex organs of the floor of the lateral ventricles, and which are curved in the manner of a vault.‖‖

---

* *De Basi Encephali*, tab. iii.

† *Abhandlung vom Gehirne*, Taf. iv.

‡ *Traite d'Anat. et de Physiol.* pl. xxv.

§ *Anat. et Physiol. du Cerveau*, pl. xi.

‖ *Trait. d'Anat. et de Phys.* pl. xx. xxiv.

¶ *Encefalotomia*, P. ii. No. 47.

** *De Penitiore Structura Cerebri*, p. 69.

†† *Anatomia*, c. 14, in 16mo. Harderw, 1658.

‡‡ *Observations on the Nervous System*, tab. iii. F.

§§ GALL, however, (*Anat. et Phys. du Cerveau*, i. 296, 514,) has not perceived this hole.

‖‖ MALACARNE (*Encefal.* P. ii. No. 54,) does not recognize the figure of a vault in the human subject.

It rests on four pillars, two anterior and two posterior: the first descend perpendicularly; the second run backwards, and separate as they descend. From this disposition it arises that the vault is narrow in front and broad behind.

D. *The pillars and mammillary eminences.*—The anterior pillars descend parallel to each other behind the anterior commissure; they become engaged in the inferior substance of the cerebrum,* traverse it, and re-appear at its base, where they terminate in the *mammillary eminences.* The latter are two pisiform tubercles, situate at the base, and before the crura, of the cerebrum: this substance is white, slightly grey internally.

On entering the vault, the anterior pillars lose their cylindrical form; they are flattened, and changed into two bands, which are attached to each other, but always in such a manner that they may be easily separated, as well as the laminæ of the septum and the two lateral portions of the corpus callosum. These bands are prolonged posteriorly, and become united, lying parallel to each other. They afterwards separate, and give origin to the posterior pillars of the vault. From this separation there results a triangular space filled by a medullary lamina, which is also a continuation of the fold of the posterior extremity of the corpus callosum, and which is marked by longitudinal lines, from which this lamina has received the name of the *lyre:* it is covered by the third choroid plexus.

The posterior pillars descend each towards the internal edge of the inferior horn of the lateral ventricle, and terminate there, gradually, about the base of the cerebrum; so that their extremity is a filament so fine, that it is termed the *festooned*, or *fringed, body;* the *bandelette*, or *tænia hippocampi.*

E. *The cornu ammonis.*—It is thus we call a medullary tuberosity, which descends in the inferior horn of the lateral ventricle, the curvature of which it follows, and which is endued with little tubercular elevations, whence it has received its name. The inferior extremity, where those inequalities are most evident, is termed the *pes leonis* or *hippocampi.* The cornu ammonis is continuous with the posterior extremity of the corpus callosum, to the angle of which it is attached. Its substance is not entirely medullary; for, when it is scraped in its longitudinal direction, it appears that this substance merely forms a thin layer, extended on the grey matter which forms the nucleus of the horn. Now, since, as we have stated in describing the inferior horn of the lateral ventricle, a convolution of the base of the cerebrum communicates with the hippocampus, we perceive that the grey substance of the base of the cerebrum is united with that of the cornu ammonis and rises upwards with it.

*Summary.*—The organs which we have just passed in review form a system in a great measure independant, and inserted between the other

---

* Sabatier (*Mem. sur quelques particularités du Cerveau, Anat.* iii. 440), and afterwards Reil (*Archiv. für die Physiol.* xi. 106), found the anterior radicles of the vault in the thalami optici, whence they descend into the mammillary eminences, and thence remount to produce the anterior pillars. We have also observed this communication between the latter and the thalami optici.

departments of the cerebrum. The vault and its posterior pillars are only placed on, and not attached to, the subjacent organs; so that this department is only fixed to the cerebral mass by the anterior pillars.

The cornu ammonis, the inferior portion of the middle lobe of the cerebrum on which it is situate, the posterior horn of the lateral ventricle, and all the posterior lobe of the cerebrum, (when the hemispheres have been removed,) appertain to the department of the corpus callosum.

This we may be assured of by the following dissection: The handle of a scalpel being glided under the posterior extremity of the corpus callosum, presents itself in the inferior horn of the lateral ventricle, below the cornu ammonis and the posterior pillar. If we then push it in the direction of the horn which traverses diagonally from behind towards the middle lobe of the cerebrum, and we tear what remains of this lobe in the direction indicated, we find that we have separated a portion composed of the posterior lobe; the inferior portion of the middle lobe, which contains the cornu ammonis, the vault, and its posterior pillars; the septum lucidum; and lastly, the corpus callosum. But this portion yet being attached by the anterior pillars and by the anterior extremity of the corpus callosum, it will be requisite to cut the pillars at the place where they are engaged in the cerebral mass, and separate the anterior extremity of the corpus callosum at the point of the beaks, and on the two sides of their attachments with the anterior lobes. In this way the entire department of the corpus callosum is detached, and there yet remain of the brain its third and fourth departments.

The department of the corpus callosum, which has now been taken away, is central, as being placed between the peripherical arrangement and that of the crura. It is very complicate, and it has no relation with the nerves, which we shall find arise from the department of the crura. Now, the nerves are the instruments by which we execute all the vital, animal, alimentary, and sexual, functions; but the intellectual functions do not demand their ministry, although they act on the cerebral substance. May, then, the department of the corpus callosum be the instrument or organ in which the intellectual functions have especially their seat? and the peripherical department, which penetrates into the interior of the cerebrum by means of the two openings at its base, and of the third opening situate behind the corpus callosum,—openings by which the choroid plexuses transmit especially to the organs of the corpus callosum their small vessels, so singularly disposed: may all this arrangement bear some relation to the corpus callosum?

[*To be continued.*]

---

### *Observations on a Mean for preventing Cancerous Degeneration in Schirrous Congestion in the Breast.* By M. le Professeur Hallé.

[From the *Nouveau Journal de Médecine*, June 1819.]

The congestions of which I speak consist in indurations, more or less considerable, comprised in the substance of the breast. Sometimes they form a round and unequal tubercle, extremely hard in its centre, round which a congestion takes place in the cellular tissue; which congestion is firmer in proportion to its nearer approach to the centres occupied by the primitive congestion. Sometimes these tubercles have been disseminated in a granular form, of the size of hemp-seed, more or less connected and grouped together, but always very hard. The surface of the skin over the seat of the tubercles is a little depressed; the sub-cutaneous tissue appears to be contracted; and the tissue even of the skin at length adheres to the centre of one or more tubercles, and becomes thinner in this place. In this point, not particularly painful on pressure from the finger, lancinating pains are felt, as if the part were pierced by an awl; they return after divers intervals, and gradually become more frequent. Firm and sensible cords often seem to extend from the seat of congestion in the breast towards the axilla, the rest of the breast remaining supple and free.

We cannot doubt, in these cases, what their termination would be sooner or later, if the disease were abandoned to itself.

Having seen many of these tumors, in circumstances which would not permit me to expect from the operation but an ephemeral success, with almost a certainty of relapse, I have resorted to the following mode of treatment:

I had a poultice made of linseed-meal, often mixed with the pulp of carrots, and then moistened with the expressed juice of carrots. The poultice having been prepared and very hot, I added to it a little lard, (half an ounce to a poultice intended to cover the breast,) to render it unctuous, and to prevent it becoming too soon cold, and drying and adhering to the skin. At the instant of its application, I covered the poultice with from half an ounce to an ounce of the powder of hemlock, which was mingled with the surface of the poultice intended to come in contact with the skin.

This poultice was kept applied during six hours in the day; it was then renewed. I had it also applied in the evening, to remain on all night. Sometimes I have it used only at night.

I have very often been content with this linseed-poultice, always moistened with lard, and covered with the cicuta powder.

The lancinating pains have constantly ceased after a few days. The congestion surrounding the hard centre, has disappeared by resolution. The centre has appeared to me to diminish in hardness and extent; sometimes it has seemed to dissipate itself; but it is well known that we must not flatter ourselves with the expectation of entirely resolving the hardness of a part that is disorganized. The progress of the disease has, however, been at least arrested, and its degenerescence, I hope, indefinitely adjourned. I could adduce six very striking examples of this success.

It has occurred to me to be consulted respecting an ulcerated cancer, established on an extensive tumor, adherent, and consequently not at all proper for the operation. The edges of the ulcer formed hard welts, and were the seats of new darting pains, which announced the further extension of ulceration. I had the poultice just described applied. It is certain that the darting pains have ceased, that the hard welts have become soft, and subsided, that the surface of the ulcer have assumed a better colour, and that the suppuration is no longer ichorous. But, as the cancer was accompanied with deep-seated pains darting into the chest, notwithstanding the amelioration of the external cancer, the internal pains have continued, and perhaps increased. I do not believe that this disease can have a fortunate termination. I have since learned that the internal disease continued its progress.

I can however assert, that, in a pulmonary affection, the progress of which was slow, in which hemoptysis to a great extent was frequently renewed, which had been preceded by the external signs of a cancerous disposition, and which was accompanied by lancinating pains, that seemed to indicate the same disease as the cause of this phthysis, the internal use of the *powder of cicuta* (and not the extract, although prepared in the manner directed by Störck,) has appeared to moderate the pains, and prolong the issue of the malady beyond the period at which it would otherwise have been fatal; that is to say, for several years, and with alleviation.

I have then, in general, advised, both externally and internally, the *powder of cicuta*, in preference to the extract. Internally, I have always given it in progressive doses, commencing from eight to twelve grains, and increasing this dose daily until it produced some degree of vertigo, which has commonly taken place when the dose has been carried to twenty grains: I then diminished the dose two grains; and I continued it in this quantity during from eight to fifteen days, again assuming the increasing progression, and constantly following the same method, to an almost indefinite extent.

I often add *camphor* to the powder of cicuta, to prevent the vertigo, and the narcotic, which are often joined with the other, effects of this remedy.

This method of employing cicuta, both externally and internally, has succeeded with me in chronic and obstinate cases of neuralgia: but the success obtained in the first attacks of these pains, is not always sustained in the recurrences of them.

As I have not regarded the use of cicuta as a new thing, since its advantages had been already stated by some illustrious practitioners, I have only spoken to my colleagues of the method which I have described, in circumstances where its employment appeared to me to be appropriate; seeing nothing remarkable in it, but the extent to which I carried its application; the perseverance I have given to it; the exclusion of other means, except those which accidents sometimes require,—as general or local bleedings; and, lastly, the preference which I gave, whether externally or internally, to the powder over the extract, however prepared; for the doses with which the sensible signs of its action are manifested, marked by vertigo, especially when it is used internally, and by the extinction of pains when applied externally, may thus be easily appreciated, and even calculated, and give to the administration of this remedy a very advantageous means of precision.

This method appears to me to have also the advantage of isolating the peculiar effects of an active, but too often neglected, and even forgotten or despised, remedy, from the want of having been employed in a proper manner.

*On the Use of the Ballota Lanata in the Cure of Dropsy.* By Dr. Rehmann, Counsellor of State, and Physician to the Emperor of Russia.

[From the *Russiche Sammlung für Naturwissenschaft und Heilkunde.*]

Medicine is indebted for a great proportion of its curative measures to popular use. Impressed with this truth, I never disdained in my voyages, and especially in that which I made in Siberia, to direct my attention to what are termed popular remedies. I have collected several; but, as all of them have not been submitted to my own experience, I shall at present confine myself to the praise of one, which may be successfully used in dropsy, as I have myself determined by observation. It is the *ballota lanata,* Lin. (*Puff-ball:—ballota,* cl. viii. ord. vi. § iii. Nat. Sys. Jussieu.) This plant, collected when its stalks, leaves, and flowers, are in full vigour, possesses such a diuretic property, that, even though there may be effusion of water in more than one cavity at the same time, the evacuation of it by the urinary passage is effected by it.

I was already convinced, in some degree, of the utility of this excellent remedy, by the statement of a respectable practitioner in Siberia, Dr. Schilling, who assured me that he cured by it all cases of dropsy not dependant on organic lesion; when I had myself an opportunity of employing this medicine.

The first trial I made with it was on a very weak subject, who, within a short time, had been attacked with typhus, then with very obstinate intermittent fever, and lastly with general dropsy. Besides a collection of fluid in the cavity of the peritoneum, there were also evident signs of hydrothorax. All the diuretics,—as squills, digitalis, &c. combined with different tonics, had been administered without success for a long time. The patient was in a state nearly approaching that of death. It was then I had recourse to the *ballota lanata,* of which I directed a decoction to be immediately administered. To excite a favourable re-action, I gave, alternately with this diuretic, thirty drops of sulphuric ether. The excretion of urine began to be increased towards the third day, which continued until all the symptoms of dropsy disappeared. I concluded the treatment, by directing the use of cinchona and some other bitters for a short time. The patient was under my observation for six months; and, although he was affected in the interval with a catarrhal fever, no signs of a recurrence of the dropsy appeared. I have since learned that she enjoys good health. In another case of abdominal dropsy, of about four months' date, I obtained the same effect.

This plant seems only to have failed in its efficacy where there has been induration of some organ or tissue; but it has constantly produced an abundant secretion of urine. The following is the way in which I administered it: Two ounces, grossly powdered, were boiled in two pints of water until this is reduced to half; half an ounce of tincture of canella was added to the strained liquor, or, according to the nature of the case, a drachm of sulphuric ether, or else from fifteen to twenty drops of tincture of opium; and half a teacup-full was taken every two hours.

The diuretic effect generally began to appear from the third to the fifth day. The urine was at first of a whitish-yellow colour, but it gradually became deeper, so that towards the seventh or eighth day it has been very dark and opaque; which seems to prove that the remedy not only has an influence on the quantity, but also on the quality, of the excreted fluid.

I should observe, that I have several times, on employing this plant, found the patient experience painful sensations in the hypochondriac regions, towards the period when the evacuation of the effused fluid was almost wholly effected; from which I have concluded, that the use of the remedy should be discontinued, or at least gradually diminished. I then only gave it in very small doses, and combined with some tonic; or else I continued the administration of it for a short time longer in the form of an infusion, taken night and morning.

---

*Abstract of a Report by* Professor DESORMAUX, *on the History of "a Case of spontaneous Cæsarean Operation from Gangrene; by* M. BERTRAND, *Surgeon at Méry-sur-Seine," presented to the Société Medicale of Paris.*

[*Bulletin de la Faculté et de la Société de Médecine de Paris*, 1819, No. iii.]

*Abstract of the History.*—E. LAURENT, 40 years of age, having arrived within a fortnight of the completion of the ninth month of her eighth pregnancy, used some powerful exertions in loading a cart with manure on the 9th of July, 1811. During this effort, she suddenly experienced very severe uterine pains, accompanied with the escape of some water, and afterwards of blood, from the vagina. The pain and hæmorrhage ceased on the patient being placed in bed. This state of calmness continued until the 15th, when the abdomen became tumefied, painful, and symptoms of the presence of inflammation were developed. No particular hardness or inequality in any part of the abdomen could be discerned. The heat of the vagina and lower part of the uterus, was somewhat different from that of the natural state. Towards the end of the month, symptoms of extreme debility succeeded this inflammatory state, and the patient seemed to be on the verge of death. At last, a red circular spot appeared between the navel and the pubes, which was soon converted into an eschar surrounded by a red circle which separated on the 13th of August, and gave vent to a large quantity of flocculent, puriform, and extremely fetid, fluid; and then to the decomposed body of a fœtus, which appeared to be of the full period.

The severity of the symptoms soon disappeared; the strength of the patient was restored; the wound gradually diminished, and towards the end of September perfectly healed. Menstruation appeared four months afterwards, and has continued regularly ever since. It should be remarked, that, on the escape of the fœtus, M. BERTRAND passed his left hand into the cavity of the uterus, which he could fully explore; and, carrying the fore-finger of the right hand into the vagina, he encountered with it that of the left, which had been directed towards the mouth of the uterus. 4

*Remarks.*—M. Bertrand thinks that the infant lost its life at the time when the violent exertion of the mother gave rise to the severe pains and the rupture of the membranes; that it remained in the uterus; and that some projecting part of its body pressing forcibly against some point of the uterus, produced inflammation of that organ, which was communicated to the adjacent parts, and terminated in the eschar comprising the paries of the uterus and the corresponding part of the abdomen.

M. Colin, of Nogent-sur-Seine, who added some notes to the history of the case, adopts this opinion, and endeavours to confirm it by arguments, and by some cases collected from various authors;* and he relates a case communicated to him by M. Haumonté, a surgeon of Bar-sur-Aube, which he considers to have some, though not a very close, relation to it. "A woman, whose pelvis was so far deformed as to render delivery in the natural way impossible, had the Cæsarean operation performed on her by M. Aubertin, with a favourable result: the wound soon healed; and she became suddenly pregnant. Matters went on well until the seventh month, when the cicatrix burst, and the fœtus presented at the wound. M. Haumonté had only to enlarge the opening, and a living infant was extracted without any effort. The woman recovered.

M. Baudlocque mentions, in a brief and general manner, a case having a close analogy to that of Haumonté. He says, "Melivier, senior surgeon of the Hôtel-Dieu at Pontoise, witnessed a case, in 1726, of a woman who could not be delivered of her thirteenth child. Sixteen days after the pains of labour, a large gangrenous ulcer appeared in the abdomen, so as to permit of the extraction of the fœtus, which had remained in the uterus; and the woman rapidly recovered."† He also refers to cases where women, carrying a dead fœtus, have become again pregnant of fœtuses, which have also perished, and which have escaped by abscesses about the navel‡ with the remains of the former fœtus.

Maurice de la Corde, in his Commentaries on the Treatise on the Diseases of Women by Hippocrates, (com. i. art. 9,) relates a case which has some analogy with that of M. Bertrand. "A peasant, in the eighth month of her pregnancy, was kicked by a horse, and thrown against the corner of a large stone, which struck her about the region of the navel. She continued all day deprived of voice, sense, and motion. Two days afterwards she was attacked with very severe febrile symptoms,—as lipothymia, vomitings, and delirium. She was purged several times, bled twice, and cooling drinks, &c. were administered. On the thirtieth day all the violence of the symptoms had diminished, and a tumor, about the size of a fist, appeared near the navel, which opened

---

* The *Histoire de l'Académie des Sciences*, an. 1709, p. 22; the *Journal de Chirurgie* of Desault, tome ii.; the *Bibliotheque Medicale* of Planque; Primrose, *De Morbis Mulierum*, p. 316; Albucasis, l. ii. c. 76; Donatus; and the *New-England Medical Journal.*

† *Recueil périodique de la Société de Medecine de Paris*, No. 22.

‡ See the last volume of this Journal, p. 217, for an analogous case, taken from a manuscript by Dr. Clarke, in the library of the British Museum.

spontaneously, and gave exit to a large quantity of pus and sanies, mingled with portions of putrefied flesh; and at length to the bones of a fœtus. After ten months the wound was completely healed, and the woman had recovered her usual health. ROUSSET* and BARTHOLIN† relate cases somewhat similar; but some of these were probably cases of extra-uterine gestation; and none of them decisively prove that the fœtus wholly remained in the uterus, and at length produced inflammation and an eschar of a portion of that organ and of the abdominal parietes, as MM. Bernard and Colin suppose, and M. Baudelocque sustained before them. Professor Desormaux is inclined to believe that rupture of the anterior part of the uterus took place in the woman Laurent, at the time when the violent pains and flow of water and blood from the vagina occurred; and that part of the fœtus passing into the abdominal cavity, caused the peritonitis, the formation of purulent matter, and the eschar of the abdominal parietes; and he also thinks, that the edges of the opening in the uterus having been affected with gangrene, as ordinarily takes place in such cases, the cavity of the uterus and the accidental cavity formed between that organ and the abdominal paries may be formed into one.

---

### Medical and Philosophical Intelligence.

* *Traité de l'Hystérotomotokie*, p. 125.

† *De Insolitis partûs Humani Viis.*

